# In idiopathic scoliosis distances of spinal cord to thoracic pedicle are within 2 mm in a large region of the thoracic apex

**DOI:** 10.1038/s41598-024-64971-z

**Published:** 2024-06-21

**Authors:** Joost A. Burger, Luis Becker, Zhao Li, Zhen Wang, Hendrik Schmidt, Matthias Pumberger, Friederike Schömig

**Affiliations:** 1 Charité-Universitätsmedizin Berlin, Center for Musculoskeletal Surgery, Charitéplatz 1, 10117 Berlin, Germany; 2 Julius Wolff Institute, Berlin Institute of Health at Charité-Universiätsmedizin Berlin, Berlin, Germany

**Keywords:** Idiopathic scoliosis, Spinal cord, Thoracic spine, Screw misplacement, Apex vertebra, Neurosurgery, Magnetic resonance imaging

## Abstract

Despite a 15% misplacement rate of screws in idiopathic scoliosis surgery, little is known about the relationship between pedicles and nerve structures in the entire thoracic curve. This study aimed to explore the spinal cord’s proximity to the pedicle wall at each thoracic vertebra in the entire thoracic curve, while considering different anatomical changes. Spinal cord to medial pedicle distances were measured on magnetic resonance imaging in 73 patients who underwent posterior spinal fusion with pedicle screw instrumentation. Associations with different variables were examined. A total of 51 patients (69.9%) showed a distance within 2 mm at the apex vertebra on the concave side, more than 50% had a distance within 2 mm in the next thoracic vertebra level above and below, and more than 25% two levels above and below. Weak correlations were found between proximity of the spinal cord at the apex vertebra and vertebra’s level and Cobb angle on the concave side (r = − 0.310, P = 0.008, r = 0.380, P = 0.001, respectively). These results emphasize the importance of thorough assessment when placing thoracic pedicle screws in idiopathic scoliosis patients. Further research is warranted to develop surgical strategies aimed at preventing potentially neurological complications resulting from screw misplacement.

## Introduction

Instrumentation with pedicle screws is the preferred method for treating scoliotic deformities. These surgical interventions demand precise pedicle screw placement to achieve favorable outcomes. The fundamental role of accurate screw positioning within the vertebral pedicles becomes magnified in this context, as it determines the success of spinal correction while minimizing the potential risks of damaging visceral, vascular, or neurologic structures.

Screw misplacement has been reported to be the most common complication of thoracic pedicle screws^1^. The average rate of screw misplacement in patients with scoliosis can be as high as 15.7% when evaluated by postoperative computed tomography (CT) imaging^1^. Handling screws in the thoracic spine within scoliotic deformities presents challenges due to the small pedicle dimensions, wide variation in morphologic pedicle features and the little space between the medial pedicle wall and the spinal cord on the concave side of the curve’s apex^2,3^. A slight screw deviation from the intended trajectory can lead to catastrophic consequences with relation to the spinal cord^4,5^. As such, some authors have suggested that any misplaced thoracic pedicle screw within the spinal canal should be removed due to the potential risk of early or late neurological complications^6^. The proximity of the spinal cord can vary significantly due to the complex anatomical alterations in these patients. Scoliosis involves lateral curvature of the spine, often accompanied by vertebral rotation with a tendency to flatten the normal kyphosis of the thoracic spine^7^. Several studies have consistently demonstrated a relationship between lateral curvature and the proximity of the spinal cord to the pedicle wall on the concave side, with particular focus on investigating this relationship only at the level of the thoracic apex^2,8^. However, there is a noticeable scarcity of research exploring how this distance variate throughout the whole thoracic curve^8^. Such investigations are crucial for surgeons to gain a comprehensive understanding of the expected distances at multiple levels, aiding in surgical planning. Moreover, little attention has been paid to the influence of additional variations such as vertebral body rotation, pedicle angle and thoracic kyphosis angles on the proximity of the spinal cord to the pedicle wall.

With the aforementioned knowledge, the primary aim of this study was to investigate the proximity of the spinal cord to the pedicle wall at each thoracic vertebra within the whole thoracic curve, considering various anatomical alterations. We hypothesized that the proximity of the spinal cord will gradually decrease on the concave side while extending below and above the thoracic apex spine body. Furthermore, we hypothesized that the strongest correlation exists between spinal cord proximity and lateral curvature, while correlations with vertebral body rotation, pedicle angle, and thoracic kyphosis angle are weaker.

## Materials and methods

This retrospective study received institutional ethical approval (EA2/049/22) from the Institutional Ethics Board of Charité-Universitaetsmedizin Berlin and was performed using data from a single academic medical center. Patient informed consent was waived due to the retrospective study design. All investigations were performed in compliance with the applicable legal requirements.

All patients with idiopathic scoliosis who underwent posterior spinal fusion using pedicle screw instrumentation between 2011 and 2021 and had preoperative radiographic and MRI examinations were included. The indication for a posterior spinal fusion was given if the curve’s Cobb angle exceeded 40° with ongoing curve progression and the patient had reached skeletal maturity. Exclusion criteria were prior spinal surgery or incomplete imaging data (lack of preoperative MR imaging, full spinal radiographs, and bending radiographs).

All patients were classified according to the Lenke classification using full spinal and bending radiographs^9^. Structural curvatures were defined as persisting curvatures over 25° Cobb angle in bending radiographs. Compensatory curvatures were defined as redressable curvatures of less than 25° Cobb angle.

**Figure 1:**
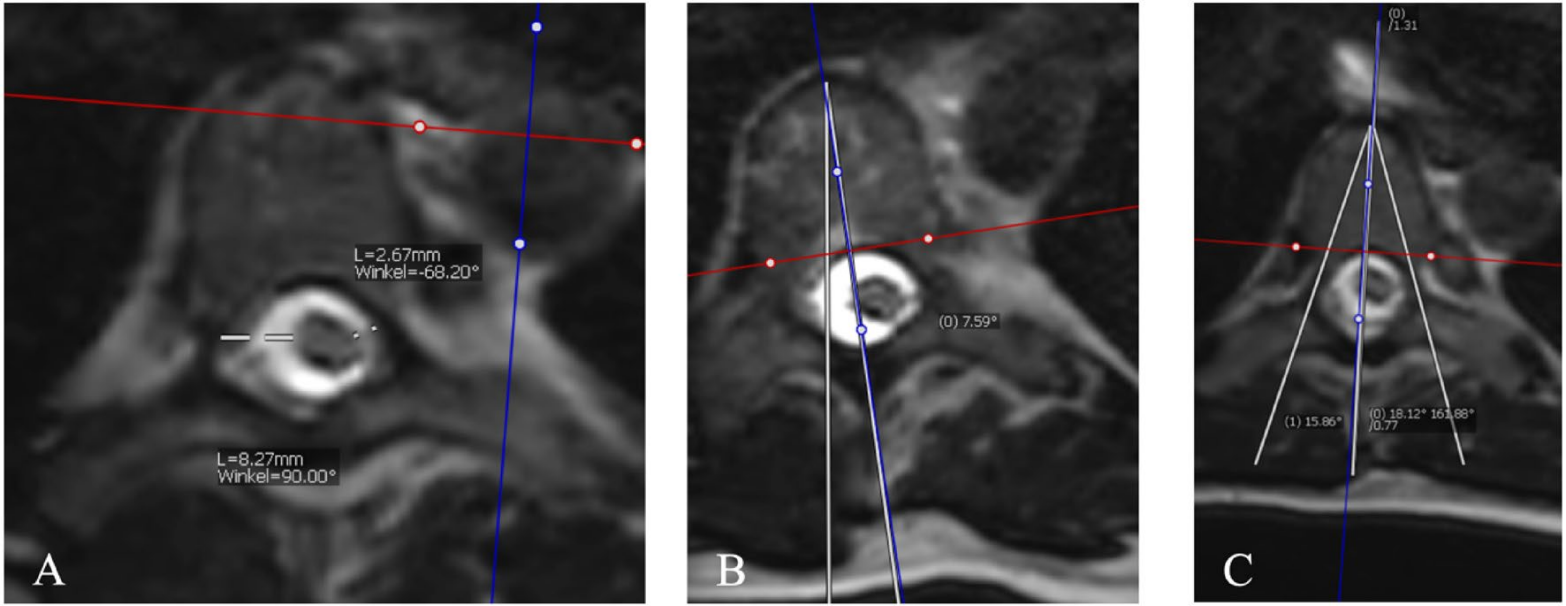
Example of the cross-sectional area measurement. (**A**) Pedicle to spinal cord distance measurement. (**B**) Vertebral body rotation measurement. (**C**) Pedicle angle measurement.

The MR images were used to take axial measurements of the vertebrae. Prior to measurement, the slicing plane was adjusted to align with the superior endplate of the respective vertebrae in both the sagittal and coronal planes. This alignment ensured to obtain a true axial slice of the vertebrae. The shortest distance from the spinal cord to the medial pedicles of the concave and convex side at the curve’s apex in the axial view was measured (Fig. 1A). Additionally, all vertebrae within the thoracic curve were measured. The vertebral body rotation and pedicle angle were determined in the axial view as well. The vertebral body rotation was similar to that described by Aaro and Dahlborn and the pedicle angle similar to that described by Berry et al., (Fig. 1B and C)^10,11^. Thoracic Cobb angles, thoracic kyphosis angles and upper body length were determined using preoperative standing EOS images of the spine. All radiological measurements were performed using the Phönix-PACS software (Phönix-PAXS GmbH, Freiburg im Breisgau, Germany) by one trained investigator (*blinded*). To test inter- and intra-observer reproducibility of the radiological measurements, a second trained investigator (*blinded*) scored a subset of 20 randomly selected patients twice, with an interval of 1 week in between. The intraclass correlation coefficients (ICCs) was used to determine the inter- and intra-observer reproducibility. ICCs for all radiographical measurements were considered to be good to excellent (Table 1).

**Table 1:** The inter- and intra-reproducibility of radiological measurements using the intraclass correlation coefficients (ICCs) with 95% Confidence Interval (CI).

Radiographical measurements	Inter-reproducibility	Intra-reproducibility
Thoracic Cobb-angle	0.988 (0.971–0.955)	0.994 (0.986–0.998)
Thoracic kyphosis angle	0.976 (0.937–0.991)	0.997 (0.991–0.999)
Upper body length	0.903 (0.752–0.963)	0.997 (0.993–0.999)
Pedicle to spinal cord distance on concave side	0.840 (0.446–0.944)	0.946 (0.863–0.979)
Pedicle to spinal cord distance on convex side	0.757 (0.391–0.903)	0.895 (0.594–0.964)
Vertebral body rotation	0.978 (0.945–0.991)	0.976 (0.939–0.990)
Pedicle angle on concave side	0.973 (0.931–0.989)	0.971 (0.927–0.988)
Pedicle angle on convex side	0.983 (0.956–0.994)	0.972 (0.931–0.989)

### Statistical analysis

Normally distributed continuous data were presented as the mean, standard deviation and range. Medians with interquartile ranges (IQR) were used when data were skewed. Frequencies and percentages were used to present discrete variables. The Student t test was used for comparisons of means between the convex and concave side and the chi-square test for comparisons of frequencies. Pearson’s correlation coefficients were calculated, depending on the level of data, and scatter-plots were used to examine any associations with the distance of the medial pedicle to spinal cord of the apex vertebra prior to the multiple regression analysis. To avoid multicollinearity among predictors, a collinearity diagnostics procedure was computed for all the independent variables before regression analysis. All statistical analyses were performed using SPSS software Version 25 (SPSS, Armonk, New York, USA) with statistical significance set at P < 0.05.

## Results

We identified 100 patients who met the inclusion criteria, four of which were excluded due to prior spinal surgery, and 24 due to missing imaging data. A total of 73 patients with MR images were available.

There were 59 females (80.8%) and 14 males (19.2%) with a median age of 16.0 years (IQR, 14.0–18.0 years). Median height was 163.0 cm (IQR, 159.3–170.0 cm) and median weight was 54.0 kg (range, 45.0 to 62.0 kg) with a median body mass index of 19.4 (IQR, 17.4–22.3). A total of 67 patients (91.8%) had a right sided curve. Most patients were Lenke type 1 (30 patients, 41.1%) followed by type 2 (17 patients, 23.3%), type 6 (11 patients, 15.1%), type 3 (7 patients, 9.6%), type 5 (5 patients, 6.8%), and type 4 (3 patients, 4.1%). The mean thoracic Cobb angle was 58.6 ± 17.6° (range, 25.0–143.0°) and the mean thoracic kyphosis was 28.0 ± 19.7° (range, − 18.5 to 89.8°). The mean thoracic Cobb angle was 56.9° for Lenke type 1, 64.7° for type 2, 66.7° for type 3, 89.7° for type 4, 28.0° for type 5 and 53.9° for type 6. The mean upper body length of the patients was 40.1 ± 4.3 cm (range, 24.1–50.2 cm).

The thoracic curve’s apex was located at the T6 vertebra in one patient (1.4%), at the T7 vertebra in eight (11.0%), at the T8 vertebra in 26 patients (35.6%), at the T9 vertebra in 23 (31.5%), at the T10 vertebra in nine (12.3%), at the T11 vertebra in five (6.8%) and at the T12 vertebra in one patient (1.4%).

### Pedicle to spinal cord distance

**Figure 2:**
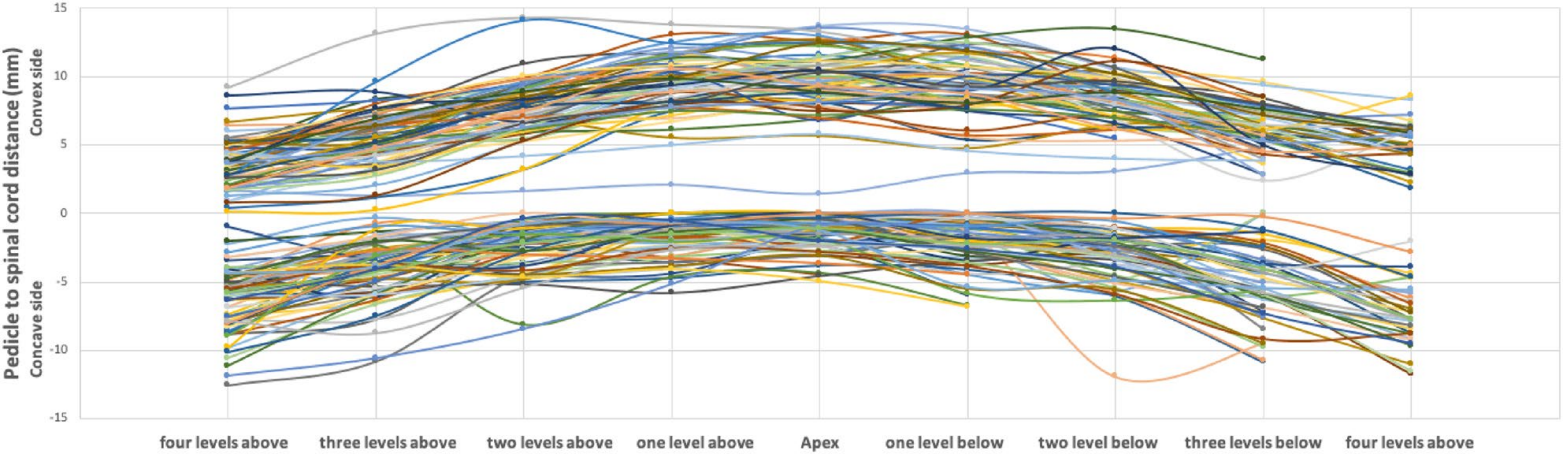
Pedicle to spinal cord distance of the concave (lower part of the graph) and convex side (upper part of the graph) throughout the Cobb angle. Patients are represented with different colors. Some lines are shorter because the vertebra lies outside the thoracic spine or the vertebrae at the upper and low limits of the curve are within a range fewer than four levels above and below the apex.

The distance of the pedicle to the spinal cord is reported in Table 2 and displayed in Fig. 2. The mean distance of the apex vertebra and the next two vertebra levels above and below on the concave side were significantly lower than on the convex side (independent t-test, p < 0.001). The difference between the mean distance became less significant three levels above (p = 0.005) and not significant three levels below the apex vertebra (p = 0.062). Conversely, the mean distance was significantly lower on the convex side than on the concave side four levels above and below the apex vertebra (p < 0.001).

**Table 2:** Pedicle to spinal cord distance (mm) at the concave and convex sides.

Variable	Concave side	Convex side	P-Value^#^
	Mean ± SD (range)	Mean ± SD (range)	
4 levels above	6.3 ± 2.3 (1.0–12.6)	3.7 ± 1.8 (0.1–9.2)	< 0.001
3 levels above	4.1 ± 2.1 (0.3–10.8)	5.4 ± 2.1 (0.3–13.4	0.005
2 levels above	2.6 ± 1.7 (0.0–8.4)	7.6 ± 2.1 (1.6–14.3)	< 0.001
1 level above	1.8 ± 1.4 (0.0–5.8)	9.5 ± 2.0 (2.1–13.8)	< 0.001
Apex vertebra	1.5 ± 1.3 (0.0–4.9)	9.7 ± 2.1 (1.4–13.7)	< 0.001
1 level below	2.1 ± 1.7 (0.0–6.8)	9.4 ± 2.1 (2.9–13.5)	< 0.001
2 levels below	3.1 ± 1.9 (0.0–11.9)	8.4 ± 1.8 (3.1–13.5)	< 0.001
3 levels below	5.4 ± 2.4 (0.3–11.9)	6.3 ± 1.7 (2.4–11.3)	0.062
4 levels below	7.2 ± 2.3 (2.0–11.8)	5.1 ± 1.6 (1.9–8.6)	< 0.001

SD, standard deviation.

^#^A p-value of < 0.05 represents a significant difference.

Categorized distances are reported in Table 3. A total of 51 patients (69.9%) showed a distance within 2 mm on the concave side for the apex vertebra with a gradually decrease of the number of patients for every next level above and below the thoracic apex vertebra. On the convex side, one patient showed a distance within 2 mm at the apex vertebra. Moreover, with each successive vertebral level above the apex vertebra, there was a gradual increase in the number of patients displaying a distance within 2 mm on the convex side. The percentage of patients with a distance within 2 mm four levels above the apex vertebra was higher on the convex side (19.2%) compared to the concave side (2.7%).

**Table 3:** Categorized pedicle to spinal cord distance at the concave and convex side.

	0–2 mm	2–4 mm	4–6 mm	> 6 mm	N/A
	N (%)	N (%)	N (%)	N (%)	N (%)
Concave side					
4 levels above	2 (2.7)	6 (8.2)	31 (42.5)	33 (45.2)	1 (1.4)
3 levels above	11 (15.1)	27 (37.0)	24 (32.9)	10 (13.7)	1 (1.4)
2 levels above	33 (45.2)	23 (31.5)	14 (19.2)	2 (2.7)	1 (1.4)
1 level above	49 (67.1)	18 (24.7)	6 (8.2)	0 (0.0)	0 (0.0)
Apex	51 (69.9)	18 (24.7)	4 (5.5)	0 (0.0)	0 (0.0)
1 level below	40 (54.8)	22 (30.1)	8 (11.0)	2 (2.7)	1 (1.4)
2 levels below	20 (27.4)	29 (39.7)	14 (19.2)	3 (4.1)	7 (9.6)
3 level below	5 (6.8)	10 (13.7)	22 (30.1)	21 (28.8)	5 (6.8)
4 levels below	0 (0.0)	3 (4.1)	7 (9.6)	24 (32.9)	39 (53.4)
Convex side					
4 levels above	14 (19.2)	31 (42.5)	21 (28.8)	6 (8.2)	1 (1.4)
3 levels above	4 (5.5)	12 (16.4)	26 (35.6)	30 (41.1)	1 (1.4)
2 levels above	1 (1.4)	2 (2.7)	10 (13.7)	59 (80.8)	1 (1.4)
1 level above	0 (0.0)	1 (1.4)	2 (2.7)	70 (95.9)	0 (0.0)
Apex	1 (1.4)	0 (0.0)	2 (2.7)	70 (95.9)	0 (0.0)
1 level below	0 (0.0)	1 (1.4)	5 (6.8)	66 (90.4)	1 (1.4)
2 levels below	0 (0.0)	2 (2.7)	2 (2.7)	62 (84.9)	7 (10.3)
3 level below	0 (0.0)	6 (8.2)	20 (27.4)	32 (43.8)	5 (6.8)
4 levels below	1 (1.4)	6 (8.2)	15 (20.5)	12 (16.4)	39 (53.4)

N/A: not available because the vertebra lies outside the thoracic spine or the vertebrae at the upper and low limits of the curve are within a range fewer than four levels above and below the apex.

### Correlation between distance and other parameters

**Figure 3:**
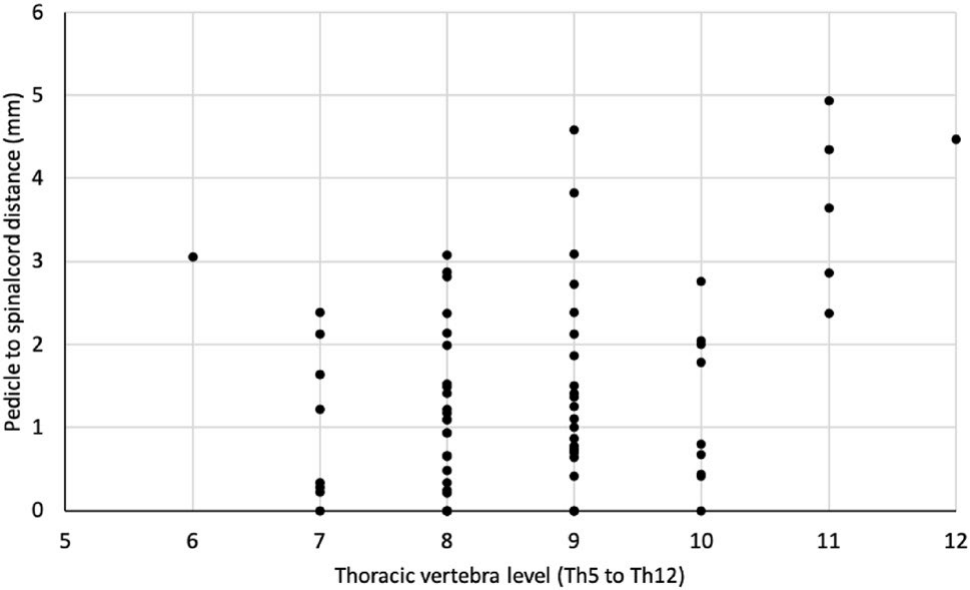
Pedicle to spinal cord distance by thoracic vertebra on the concave side.

**Figure 4:**
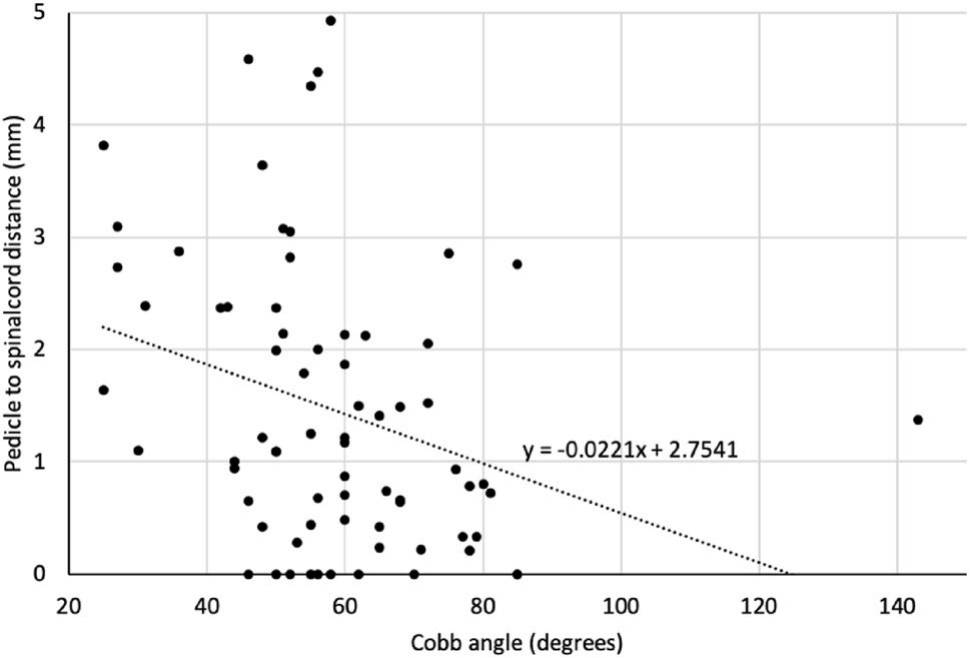
Pedicle to spinal cord distance by Cobb angle (degrees) on the concave side.

On the concave side, there was a weak but significant correlation between the distance of the pedicle to the spinal cord and the apex level (Pearson r = 0.380, P = 0.001, Fig. 3). Distances within 2 mm were seen for Th7 to Th10, but not for Th6, Th11 or Th12. A significant negative correlation was found between the distance and the Cobb angle (Pearson r = − 0.310, P = 0.008, Fig. 4) on the concave side. Two patients with a Cobb angle less than 40° showed a pedicle to spinal cord distance of less than 2 mm.

No significant correlation was found between the distance of the pedicle to the spinal cord and the thoracic kyphosis angle, pedicle angle, vertebra rotation or upper body length on the concave side (Pearson r = 0.180, P = 0.136, r = -0.132, P = 0.26, r = − 0.212, P = 0.072, r = − 0.047, P = 0.700 respectively). On the convex side, the only significant correlation was found between distance and Cobb angle (Pearson r = 0.232, P = 0.048).

There was a significant difference in the number of patients with a pedicle to spinal cord distance less than 2 mm at the apex vertebra on the concave side between Lenke types (Table 4). After excluding the Lenke type 5 cases from the analysis, there was no significant difference left (P = 0.180). No differences were found on the convex side.

**Table 4:** Number of patients (%) with pedicle to spinal cord distance less than 2 mm at apex vertebra by Lenke type.

	Lenke Type						P-value‡
	1	2	3	4	5	6	
Total	30	17	7	3	5	11	
Concave side							*0.033*
$\le$ 2.0 mm	24 (80.0)	13 (76.5)	5 (71.4)	3 (100)	1 (20.0)	5 (45.5)	
> 2.0 mm	6 (20.0)	4 (23.5)	2 (28.6)	0 (0.0)	4 (80.0)	6 (54.5)	
Convex side							0.918
$\le$ 2.0 mm	1 (3.3)	0 (0.0)	0 (0.0)	0 (0.0)	0 (0.0)	0 (0.0)	
> 2.0 mm	29 (96.7)	17 (100)	7 (100)	3 (100)	5 (100)	11 (100)	

Significant values are in [italics].

^‡^Chi-Square test. A P-value of < 0.05 represents a significant difference.

The results of the multivariable regression analyses showed that the level of the apex vertebra and the Cobb angle were two independent predictors for the distance of the medial pedicle to the spinal cord (Table 5). The analysis of the collinearity tolerance test showed that the tolerance value was not close to zero, indicating no collinearity.

**Table 5:** Multivariable analysis using a linear regression model with pedicle to spinal cord distance of the apex vertebra at the concave side as the dependent variable.

Variable	Coefficient (95% CI)	Standard Error	P-Value^#^	C-value*
Cobb angle	− 0.024 (− 0.039, − 0.009)	0.007	*0.002*	0.996
Vertebra level	0.433 (0.212, 0.655)	0.111	< *0.001*	0.996

Significant values are in [italics].

C-value, collinearity tolerance value; CI, confidence interval.

^#^A p-value of < 0.05 represents a significant regression coefficient.

*A C-value close to zero, indicates collinearity.

## Discussion

The main finding was that a considerable number of patients with idiopathic scoliosis exhibited spinal cord distances to the medial pedicle within two millimeters across a wide area of the curve’s concave side. More than 70% of the patients had a distance within 2 mm at the thoracic apex level on the concave side, more than 50% one level above and below the apex vertebra and more than 25% two levels above and below the apex vertebra. The percentage of patients with a distance within 2 mm shifted at the upper level of the thoracic curve, transitioning from a higher number of patients on the concave side to a higher number on the convex side. Additionally, as the Cobb angle increased, there was a correlation with the distance of the medial pedicle to the spinal cord of the apex vertebra decreasing on the concave side and increasing on the convex side.

While the rate of malpositioned pedicle screws is reported at 4.2%, neurological issues stemming from incorrect screw placement during the surgical treatment of idiopathic scoliosis have been found to occur in 1.8% of patients^1,12^. However, the findings of the study by Mac-Thiong et al.^6^ suggest that a delayed neurological deficit can potentially arise from screw misplacement within the spinal canal, even in patients who are neurologically intact immediately after surgery. As several studies only assess early complications after idiopathic scoliosis, it is possible that late neurological deficits of misplaced screw are underreported in the current literature. A case presented by Papin et al.^5^ highlighted unusual disruptions caused by spinal cord compression due to the penetration of two screws (located at T8 and T10) by 4 mm. The resulting symptoms, which included epigastric pain, tremors in the right foot while at rest, and abnormal sensations in the legs, necessitated a revision surgery to replace the screws. Full recovery was observed one month after the surgical intervention.

The occurrence of thoracic screws being positioned medially is rather frequent, though the extent of this phenomenon and its acceptability remain subjects of debate. It is generally well tolerated when the medial wall penetration does not exceed 2 mm. Gertzbein and Robbins conducted an assessment of the precision of pedicle-screw placement in forty consecutive patients predominantly treated for spine fractures, spanning from the tenth thoracic vertebra to the fourth lumbar vertebra^13^. Surprisingly, they discovered that even when a screw had penetrated the inner border of the pedicle by up to 4 mm, no early neurological symptoms were observed. In this range of 0–4 mm, they characterized it as a safety zone. Others have proposed different “safe zone” thresholds based on cohorts including patients with scoliosis^14–16^. Kim et al.^14^ indicated a “definite safe zone” within 2 mm, a “probable safe zone” between 2 and 4 mm, and a “questionable safe zone” encompassing 4–8 mm of medial encroachment. However, in patients with idiopathic scoliosis, the present study showed as expected that the spinal cord is shifted towards the side of concavity, placing the neural structures in close proximity to the medial pedicle wall.

The present study showed that the small distance (< 2 mm) from the medial pedicle wall to the spinal cord is not limited to the apex vertebra on the concave side, but also applies to several thoracic levels above and below the apical level within the Cobb angle. Comparable findings were found by Liljenqvist et al.^2^, showing that the average width of the epidural space was < 1 mm at the thoracic apical level on the concave side, however, not considering to which extent above and below the thoracic apical level. An interesting finding of the current study was the high prevalence of patients with a distance within 2 mm four levels above the thoracic apex on the convex side. This phenomenon is likely attributed to the change in curvature direction proximal to the thoracic curve, resulting in a shift of the spinal cord more to the convex side at the end vertebral level of the thoracic curve. Taken together, the distance gradually decreases on the concave side while extending below and above the thoracic apex, with an increased risk of small distances on the convex side in the upper vertebra level of the thoracic curve.

While studies involving MRI have shown a relationship between the proximity of the spinal cord and the degree of lateral curvature at the apex level, currently little effort has been made to evaluate the relationship between other anatomical alterations and pedicle to spinal cord distance in patients with idiopathic scoliosis. The present study revealed a limited correlation with vertebra rotation, pedicle angle, kyphosis angle, and upper body length, suggesting their clinical relevance may be limited. Despite the stronger correlation of the Cobb angle, surgeons should be aware that distances within 2 mm can exist not only for large Cobb angles but even for small Cobb angles (< 40°).

When interpreting the differences among various Lenke types in the current study, caution is warranted due to the limited sample sizes. As expected, lower percentage of patients exhibited a distance of less than 2 mm between the medial pedicle and spinal cord on the concave side for Lenke types 5 and 6, attributed to the smaller Cobb angle of the thoracic curvature in contrast to Lenke types 1 to 4. Despite the small sample size, including different Lenke types sheds light on understudied types, offering valuable insights and encouraging the generation of future research directions.

In the past few years, modern intraoperative navigation techniques have been increasingly used for more accurate pedicle screw placement. It has been shown that their use results in a decrease in pedicle breach rates while the effect on neurological complication rates remains debated^17^. Regarding our study’s results showing very small distances between the medial pedicle wall and the spinal cord at the curve’s apex, the use of intraoperative navigation needs to be taken into consideration even more as it allows more accurate screw placement. Even though the neurological complication rate is low, considering that the patients treated are otherwise healthy children, every complication needs to be avoided if possible.

The strength of this study is that it demonstrates the relationship between the distance from the spinal cord to the medial wall of the pedicle throughout the thoracic curve, considering various anatomical alterations. However, the present study also has limitations. Several patients had to be excluded due to missing imaging data, thus rendering smaller patient groups for the subgroup analyses. Furthermore, due to our study’s retrospective design, MRIs were not performed with a standardized protocol.

In conclusion, the findings showed a significant number of patients with a spinal cord distance within two millimeters of the medial pedicle across a wide range of the thoracic curve on the concave side, with a notable shift in proximity at the upper level of the thoracic curve from the concave to the convex side. This suggest that thoracic pedicle screw placement should be evaluated carefully throughout the curvatures of the thoracic spine in patients with idiopathic scoliosis. Even though neurological deficits due to pedicle malpositioning are rare, strategies to avoid this devastating complication need to be further investigated.

## Data Availability

The datasets generated and analyzed during the current study are available from the corresponding author on reasonable request.
